# Joint spatio-temporal modelling of adverse pregnancy outcomes sharing common risk factors at sub-county level in Kenya, 2016–2019

**DOI:** 10.1186/s12889-021-12210-9

**Published:** 2021-12-30

**Authors:** Julius Nyerere Odhiambo, Benn Sartorius

**Affiliations:** 1 Discipline of Public Health Medicine, College of Health Sciences, University of KwaZulu-Natal, 2nd Floor George Campbell Building, Howard College Campus, Durban, 4001 South Africa; 2 Department of Management Science and Technology, The Technical University of Kenya, Nairobi, Kenya; 3 Ignite Lab, Global Research Institute, William and Mary, Williamsburg, Virginia, USA; 4 Centre for Tropical Medicine and Global Health, Nuffield Department of Medicine, University of Oxford, Oxford, UK; 5 Department of Health Metrics Sciences, University of Washington, Seattle, USA

**Keywords:** Mapping, Risk, Low birth weight, Pre-term birth, Stillbirths, Neonatal deaths, Bayesian shared component, Kenya

## Abstract

**Background:**

Adverse pregnancy outcomes jointly account for a high proportion of mortality and morbidity among pregnant women and their infants. Furthermore, the burden attributed to adverse pregnancy outcomes remains high and inadequately characterised due to the intricate interplay of its etiology and shared set of important risk factors. This study sought to quantify and map the underlying risk of multiple adverse pregnancy outcomes in Kenya at sub-county level using a shared component space-time modelling framework.

**Methods:**

Reported sub-county level adverse pregnancy outcomes count from January 2016 – December 2019 were obtained from the Kenyan District Health Information System. A Bayesian hierarchical spatio-temporal model was used to estimate the joint burden of adverse pregnancy outcomes in space (sub-county) and time (year). To improve the precision of our estimates over time and space, information across the outcomes were combined via the shared and the outcome-specific components using a shared component model with spatio-temporal interactions.

**Results:**

Overall, the total number of adverse outcomes in pregnancy increased by 14.2% (95% UI: 14.0–14.5) from 88,816 cases in 2016 to 101,455 cases in 2019. Between 2016 and 2019, the estimated low birth weight rate and the pre-term birth rate were 4.5 (95% UI: 4.4–4.7) and 2.3 (95% UI: 2.2–2.5) per 100 live births. The stillbirth and neonatal death rates were estimated to be 18.7 (95% UI: 18.0–19.4) and 6.9 (95% UI: 6.4–7.4) per 1000 live births. The magnitude of the spatio-temporal variation attributed to shared risk was high for pre-term births, low birth weight, neonatal deaths, stillbirths and neonatal deaths, respectively. The shared risk patterns were dominant in sub-counties located along the Indian ocean coastline, central and western Kenya.

**Conclusions:**

This study demonstrates the usefulness of a Bayesian joint spatio-temporal shared component model in exploiting specific and shared risk of adverse pregnancy outcomes sub-nationally. By identifying sub-counties with elevated risks and data gaps, our estimates not only assert the need for bolstering maternal health programs in the identified high-risk sub-counties but also provides a baseline against which to assess the progress towards the attainment of Sustainable Development Goals.

**Supplementary Information:**

The online version contains supplementary material available at 10.1186/s12889-021-12210-9.

## Background

Adverse pregnancy outcomes (APOs) are a major cause of neonatal mortality and morbidity and affects millions of women of childbearing age worldwide and is a critical global health concern. While large strides have been made in tracking progress towards the attainment of global maternal health targets in Sub-Saharan Africa (SSA), the World Health Organization (WHO) estimates that approximately 810 women die each day as a result of preventable causes associated with pregnancy and childbirth [1–4]. Despite the global priority and substantial investment in maternal and child health programs in recent decades [5], the burden of APOs remains unacceptably high in SSA and Southern Asia, with both regions accounting for approximately 86% of the global maternal deaths [1]. Recent estimates of APOs have been 546 maternal deaths per 100,000 live births, 25.9 neonatal deaths per 1000 live births and 13.9 stillbirths per 1000 total births [6–8]. These sobering trends are mirrored in Kenya, which risks missing out on the 2030 neonatal mortality target of 12 deaths per 1000 live births given its high neonatal mortality rate of approximately 19.6 deaths per 1000 live births and 11.5 low birth weight babies per 100 live births reported in 2018 [9]. To address this growing burden, national initiatives such as free maternity services, elimination of user fees for public primary health care and the beyond zero campaign have been established to improve pregnancy outcomes and increase institutional delivery rates [10].

As a step towards renewing its focus towards maternal and child health programs and achieving global health targets, the United Nations Sustainable Development Goals (SDGs) set ambitious health targets for mothers, infants and children. Target 3.1 seeks to reduce maternal mortality ratio to less than 70 per 100,000 live births. In contrast, target 3.2 aims to end preventable neonatal mortality to 12 per 1000 live births and sustain universal healthcare by 2030 [11]. Moreover, the WHO conceptualised two global initiatives namely; Ending Preventable Maternal Mortality (EPMM) [12] and the Every Newborn Action Plan (ENAP) [13]. These initiatives sought to catalyse global action into addressing disparities in maternal health outcomes among sub-populations. These ambitious targets were further entrenched by two of the six global nutritional targets for 2025 [14], which sought to achieve; a 50% reduction of maternal anaemia and a 30% reduction of low birth weight (LBW) as vital components for improving maternal health.

The government of Kenya strives to achieve universal health coverage for its population and alleviate the burden imposed by adverse pregnancy outcomes in women of childbearing age. Mapping sub-county level estimates would provide a greater understanding of the variability in spatio-temporal patterns of adverse pregnancy outcomes, not possible by examination of direct national estimates or by examination of direct county-level estimates [15]. This is crucial for; monitoring progress towards global maternal health priorities and orienting national policies to identify high burden sub-counties and high-risk populations [11, 15, 16].

Sub-nationally, little is also known about the joint and shared risk estimates of adverse pregnancy outcomes and their association with related health outcomes across the continuum of maternal health care. This may be attributed to the complex interplay of risk factors that remains inadequately characterised in the spatial and temporal domain [17–19]. This may be attributed to the paucity of data on individual pregnancy outcomes and risk factors sub-nationally. This becomes more apparent when data is stratified at policy-relevant spatial scales to accommodate different demographic dimensions. To stabilise estimates, strengthen inference occasioned by data scarcity, we borrow information across adjacent sub-counties, diseases/health outcomes and sub-populations with common risk factors. Previous studies have demonstrated the robustness of Bayesian techniques in jointly mapping two or more diseases/health outcomes with common risk factors by leveraging information among the diseases and across neighbouring geographical units [20, 21]. To the best of our knowledge, little work on Bayesian space-time modelling has been done in maternal health research in Kenya. To quantify the underlying burden and increase epidemiologic interpretability of adverse pregnancy outcomes, we employed a Bayesian hierarchical shared component model that simultaneously accounts for both shared and specific risk components attributed to each pregnancy outcome.

## Methods

### Geographic analysis unit

**Fig. 1:**
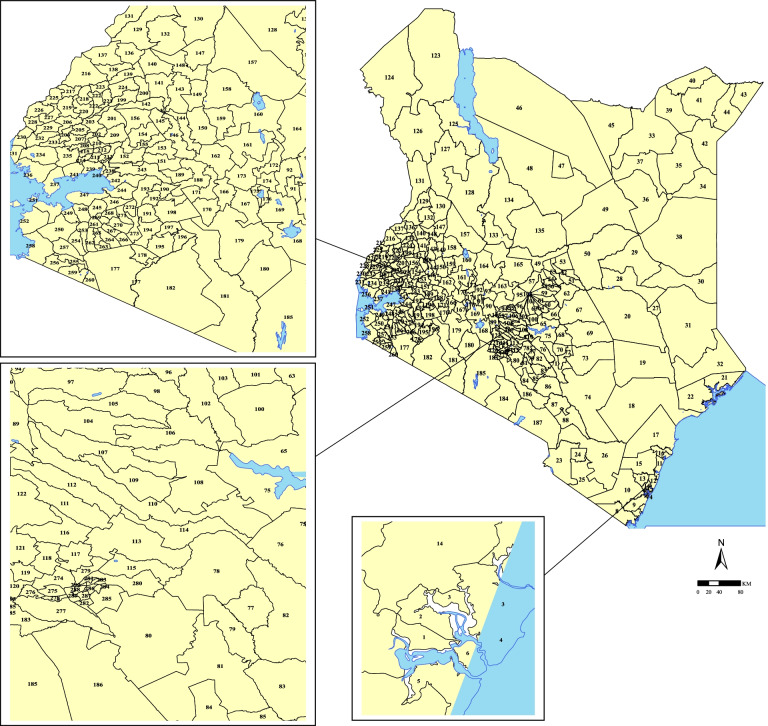
The map of Kenya showing 290 sub-counties (numbered), with the extents of major lakes and the Indian ocean is shown in light blue. The names of counties, sub-counties and their malaria endemicity status are presented in supplementary file 2. (Source: https://data.humdata.org/dataset/ken-administrative-boundaries)

Administratively Kenya is divided into 47 counties tasked with the implementation of national guidelines and policy. The counties are further subdivided into 290 sub-counties which was adopted as the unit of analysis in this study (Fig. 1).

### Kenya healthcare system

Kenya’s public health system is divided into six levels ranging from community-based care (level 1) to tertiary hospitals (level 6). Level 1 primarily consists of health promotion and awareness-raising activities at the community level; levels 2–3 include primary health care facilities, including dispensaries and health centres; and levels 4–6 include county and national referral hospitals [22, 23]. The Kenya government prioritises healthcare as a critical element in its development agenda. The Kenya Health Policy 2014–2030 seeks to achieve universal health coverage by targeting health services to regions and populations in need [16]. Additionally, in seeking to improve the facility delivery rates and reduce pregnancy-related mortality, the government abolished delivery fees in all public health facilities in 2013 [10]. This led to a significant increase in facility deliveries, with the proportion of reported births increasing from 90.1% in 2015 to 96.7% by 2019 [24]. Additionally, the devolved governance system stimulated the need for sub-national monitoring of disease burden trends and control/intervention progress. This led to a steady increase in facility reporting completeness rate (estimated to be above 90%) on key health metrics. The most recent Demographic and Health Survey (DHS) reports that only 61% of births between 2009 and 2014 were reported to have occurred in a health facility [25].

### Data assembly

Global maternal and newborn health initiatives, Every Newborn Action Plan and Ending Preventable Maternal Mortality, have identified priority indicators derived from facility-based data as important signals for monitoring progress [12, 26]. Consequently, study data was extracted from the Kenya District Health Information System (DHIS2), provided a unique opportunity/effective basis to examine the sub-national variation of APOs from 2016 to 2019 (Table 1).

**Table 1:** Definition of adverse pregnancy indicators

Data	Description	Spatial resolution	Temporal resolution
Pre-term birth	The spontaneous or the induced live delivery of a baby before 37 completed gestational weeks [27].	Sub-county	Yearly
Low birth weight	The birth weight of less than 2500 g (up to and including 2499 g) and is considered one the principal measures of birth outcomes.	Sub-county	Yearly
Stillbirth	The intrauterine death of a fetus at any time during pregnancy/Is a baby born with no signs of life at or after 28 weeks of gestation. Stillbirth rate, is consider a proxy indicator of quality of care during labour and delivery [19].	Sub-county	Yearly
Neonatal deaths	The death of a live born infant, irrespective of gestational age at birth, within the first 28 completed days of life.	Sub-county	Yearly
Live birth	The complete expulsion/extraction from its mother a of a product of conception, irrespective of the duration of the pregnancy, which, after such separation, breathes or shows any other evidence of life.	Sub-county	Yearly
ANC – 4 visits	The number of women with a live birth in a given time period that received antenatal care four or more times and is an indicator of access and use of the health care facility during pregnancy.	Sub-county	Yearly
Maternal anaemia	Is the haemoglobin concentration below 11 g per decilitre (g/dL) [28].	Sub-county	Yearly
Long-lasting insecticidal net (LLINs)	This is a core vector control intervention with an anticipated lifespan of 3 years and 20 washes based on its physical and chemical durability, that has been shown to reduce malaria illness and mortality in endemic regions [29].	Sub-county	Yearly
Intermittent preventive treatment – 2 doses (IPT2)	This a curative dose of an effective antimalarial drug (e.g. sulfadoxine-pyrimethamine) to all pregnant women during their routines antenatal care visit.	Sub-county	Yearly
Iron and Folic Acid Supplementation	Are essential micronutrient interventions used to reduce maternal anaemia, risk of low birth weight, neural tube defects in pregnancy and improve the overall pregnancy outcomes.	Sub-county	Yearly

### Selecting a suitable set variable for APOs risk

A two-step procedure prominent in mapping exercises [30, 31] was used to select the best predictors of adverse pregnancy outcomes based on their associative strength and relevance. First a non-spatial Poisson regression model was used to test the bivariate association between APOs and related risk factors. To identify the strength of each candidate covariate as a predictor and to identify the best subset of predictors for each outcome respectively. The Wald’s *p*-value, the goodness of fit statistics and the associated credible interval were then assessed. Non-collinear covariates significant at a *p*-value of < 0.05 were then included in subsequent analyses. (Supplementary file 2).

### Joint spatio-temporal shared component model

The Bayesian hierarchical spatio-temporal shared component model was used to simultaneously quantify the burden of four APOs often characterised by low counts. To improve the precision of estimates over time and space, information (similarities and differences) across the APOs was combined via the shared and the outcome-specific components using a shared component model with spatio-temporal interactions [32].

Let ***Y***_***ijk***_ be the observed number of adverse pregnancy outcomes, where ***i*** represents a given sub-county (1–290), ***j*** represents different periods (2017–2019) and ***k*** represents the different adverse pregnancy outcomes (*1 – Low birthweight, 2 – Pre-term births, 3 – Stillbirths, 4 – Neonatal deaths*).

Let ***n***_***ijk***_ be total live births for sub-county ***i*** (***i =*** **1**, …, **290**) and time period ***j*** (***j =*** **1**, …, **16**) across all the adverse outcomes ***k***(***k =*** **1**, **2**, **3**, **4**)**.**Due to the low rate of adverse pregnancy outcomes when compared to live births, and without the loss of generality, a binomial model for observed counts with a logit link function was used in the analysis.

i.e. We assume that the observed number of cases ***Y***_***ijk***_ arises from a binomial distribution, that is;

$${\boldsymbol{Y}}_{\boldsymbol{ijk}}\sim \mathbf{binomial}\left({\boldsymbol{n}}_{\boldsymbol{ijk}},{\boldsymbol{\pi}}_{\boldsymbol{ijk}}\right)\ \boldsymbol{i}=\mathbf{1},\dots, \mathbf{290};\boldsymbol{j}=\mathbf{2016},\dots, \mathbf{2019};\boldsymbol{k}=\mathbf{1},\mathbf{2},\mathbf{3},\mathbf{4}$$

Here, *π*_*ijk*_ represents the true, unknown risk in sub-county ***i*.** time ***j*** and disease ***k.***Then the proportionate rate is specified on the logit scale as:

$$Logit\left({\boldsymbol{\pi}}_{\boldsymbol{i}\boldsymbol{j}\boldsymbol{k}}\right)={\alpha}_k+{\boldsymbol{X}}_{\boldsymbol{i}}{\beta}_k+{\boldsymbol{X}}_{\boldsymbol{i}\boldsymbol{j}}{\beta}_k+{\boldsymbol{\mu}}_{\boldsymbol{i}\boldsymbol{j}\boldsymbol{k}}$$

The parameter *α*_*k*_ is the baseline risk for a given adverse pregnancy outcome, ***X***_***i***_ and ***X***_***ij***_ are the time invariant spatial covariates and space-time varying covariates (ANC– 4 visits, maternal anaemia, LLINs, IPT2, Iron, Folate) respectively. Whereas *β*_*k*_ are the vectors of regression coefficients for each pregnancy outcome that accounts for the varied risk gradients of the shared spatial and temporal components. i.e. they represent the relative weight of the contribution of the shared terms to the risk APOs. The spatio-temporal structure is defined by ***μ***_***ijk***_**,** which accounts for the possible variations of risk in the logit scale.

The joint spatial-temporal structure for each outcome was specified as follows

$${\boldsymbol{\mu}}_{\boldsymbol{i}\boldsymbol{j}\boldsymbol{k}}={\boldsymbol{\gamma}}_{\boldsymbol{k}}^{\boldsymbol{s}}{\boldsymbol{u}}_{\boldsymbol{i}}^{\boldsymbol{s}}+{\boldsymbol{u}}_{\boldsymbol{i}\boldsymbol{k}}^{\boldsymbol{s}}+{\boldsymbol{\gamma}}_{\boldsymbol{k}}^{\boldsymbol{t}}{\boldsymbol{u}}_{\boldsymbol{j}}^{\boldsymbol{t}}+{\boldsymbol{u}}_{\boldsymbol{j}\boldsymbol{k}}^{\boldsymbol{t}}+{\boldsymbol{\nu}}_{\boldsymbol{i}\boldsymbol{j}}$$

With

$$\sum \limits_{k=1}^4{\gamma}_k^s=0 \mathrm{and}\sum \limits_{k=1}^{4}\log {\gamma}_k^t=0$$

In this formulation ${u}_i^s$ are a set of common random effects associated with space, i.e. conditional autoregressive (CAR), ${u}_{ik}^s$ are outcome-specific random effects associated with space (CAR), *k* = 1, 2, 3, 4, ${u}_j^t$ are a set of common random effects associated with time, i.e. random walk of order 1 (RW1), ${u}_{jk}^t$ are outcome-specific random effects associated with time (RW1) [33–35]. The space-time interaction term/heterogeneity of order two is represented by ***ν***_***ij***_ i.e. it represents the possible variations not explained by the spatial and temporal effects in the model. A detailed description of the analyses is found in supplementary file 1.

### Computation

A two-chain Markov Chain Monte Carlo Simulation (MCMC) with 80,000 iterations and a burn-in of 4000 samples was used for estimating the model parameters. Both models were implemented in WinBUGS Version 1.4.3 (available at http://www.mrc-bsu.cam.ac.uk/bugs/welcome.shtml). ArcMap 10.7.1 was used for creating risk maps (ESRI Inc., Redlands, CA, USA).

### Model diagnostics

A random sample comprising 20% of the observed data points was drawn from the space-time cube to assess the overall model adequacy. The data with the removed points were then re-inputted into WinBUGS. The posterior estimates were then compared with the observed values. The posterior estimates (fixed effects, scaling parameters, and standard deviations of all random effects) were also assessed to check if the MCMC simulation had converged. i.e. A MC error/SD of less than 5% pointed to the model stability. Convergence of the MCMC chain was assessed visually by monitoring the trace plots for each parameter and analytically by the Gelman-Rubin statistics [36] (Supplementary file 1).

This analysis adheres to the Guidelines for Accurate and Transparent Health Estimates Reporting (GATHER) [37].

## Results

### Temporal trends in the burden of adverse pregnancy outcomes in Kenya from 2016 to 2019

**Fig. 2:**
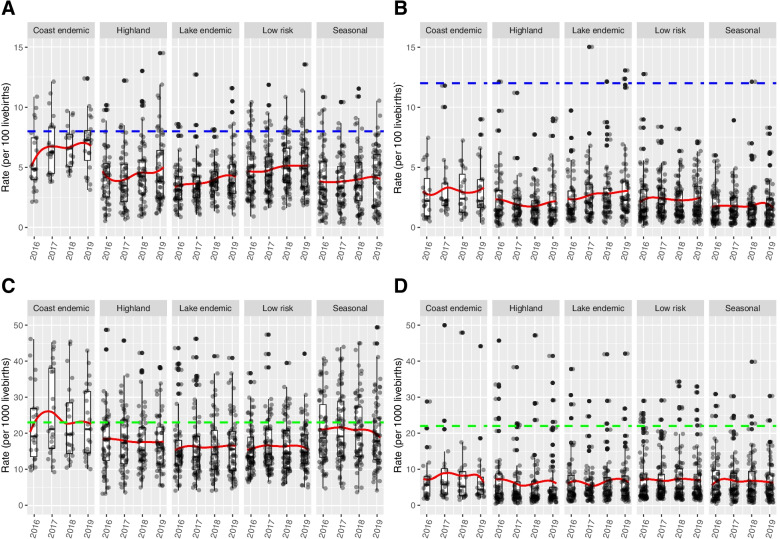
Posterior estimates (mean) of adverse pregnancy outcomes stratified by malaria endemicity from January 2016 to December 2019. **A**: Low birth weight per 100 live births, The blue dotted line (11.5 per 100 live births) represents the estimated national rate of LBW [38]. **B**: Pre-term birth rate (per 100 live births), the blue dotted line (12 per 100 live births) represents the national rate [39], **(C)**; Stillbirth rate (per 1000 live births), the green dotted line represents the estimated the national rate [8]. **D:** Neonatal death rate (per 1000 live births), the green dotted line represents the estimated national rate [40]

A total of 2,896,659 (95% Uncertainty Interval: 2,811,500 – 2,981,817) live births were reported in Kenya from January 2016 to December 2019. Overall, the total number of APOs increased by 14.2% (95% UI: 14.0–14.5) from 88,816 cases in 2016 to 101,455 cases in 2019. In particular, low birth weight increased by 19.8% (95% UI: 19.4–20.2) from 38,440 cases in 2016 to 46,051 cases in 2019 as shown in Table 2. The number of stillbirths increased by 5.5% (95% UI: 5.1–5.8) from 16,888 cases in 2016 to 17,810 cases in 2019. Pre-term births increased by 16.3% (95% UI: 15.8–16.8) from 23,597 cases in 2016 to 27,437 cases in 2019. Neonatal deaths were the least reported events and the counts slightly increased by 2.7% (95% UI: 2.4–3.0) from 9891 cases in 2016 to 10,157 cases in 2019 (Fig. 2).

**Table 2:** Temporal changes in the burden of adverse pregnancy outcomes from 2016 to 2019. Among the adverse pregnancy outcomes considered in the study, the most and least common outcomes were low birth weight and neonatal deaths, respectively

Year	2016	2017	2018	2019	Total
Low Birth Weight	38,440	23,162	43,150	46,051	150,803
Stillbirths	16,888	11,381	17,781	17,810	63,860
Pre-term births	23,597	15,672	24,976	27,437	91,682
Neonatal deaths	9891	5688	9959	10,157	35,695

### Low birth weight

**Fig. 3:**
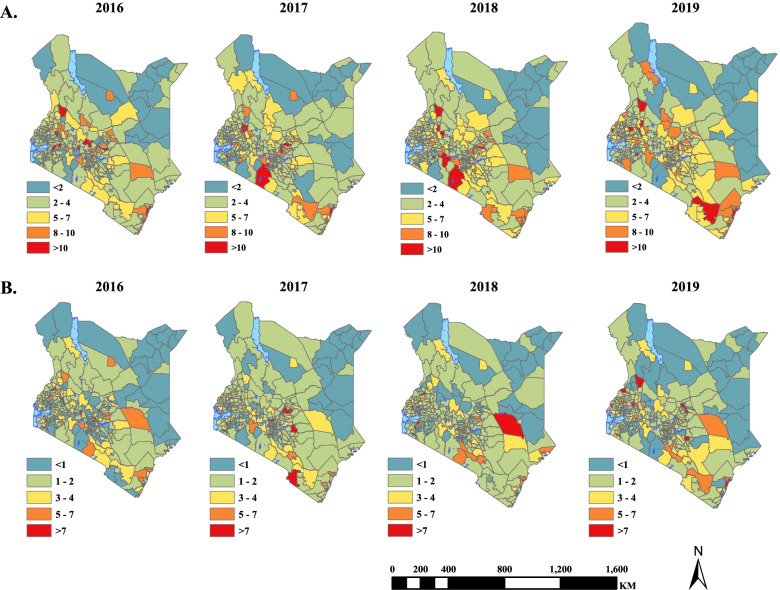
Maps showing the changing spatial evolution of the posterior median risk of (**A**) Low-birth weight per 100 live births, **B** Pre-term births per 100 live births

The low birth rate was estimated to be 4.5 (95% UI: 4.4–4.7) per 100 live births, with the proportionate risk of low birth weight increasing from 4.3 (4.0–4.7) per 100 live births in 2016 to 4.8 (4.5–5.1) per 100 live births in 2019. Our estimates suggest that 60% or 173/290 sub-counties exhibited a decline in the risk of low birth weight (ranging from an absolute difference of 7.5% in Mosop to 0.5% in Wajir South). However, the risk increased in 40% or 117/290 sub-counties (from 15.7% in Kipkelion West to 1.8% in Navakholo). Malaria endemic sub-counties along the Indian Ocean coastline exhibited the highest risk over the study period, with the highest risk of 7.8 (95% UI: 6.0–9.7) per 100 live births observed in 2018 (Supplementary file 2). Conversely, arid and semi-arid sub-counties in northern and eastern parts such as Mandera North, Mandera West, Eldas, Tarbaj, Banissa, Wajir South and Laisamis, exhibited low risk over the study period, as is shown in Fig. 3.

### Pre-term births

The estimated pre-term birth rate was 2.3 (95% UI: 2.2–2.5) per 100 live births, with 134 of 290 sub-counties exhibiting a decline in the risk pre-term births (ranging from an absolute difference of 11.6% in Olkalou to 0.01% in Borabu), while the risk increased for 156 sub-counties (ranging from to 8.3% in Jomvu to 0.02% in Gichugu) as shown in Fig. 3. Compared to other sub-counties, elevated risk was observed in a few sub-counties along the Indian Ocean coastline, whereas low risk was observed in arid and semi-arid sub-counties in the northern and eastern parts of Kenya. In 2019, elevated risk was observed in western Kenya in Lurambi, Suna East, Kisumu Central and Matayos sub-counties.

### Stillbirths

**Fig. 4:**
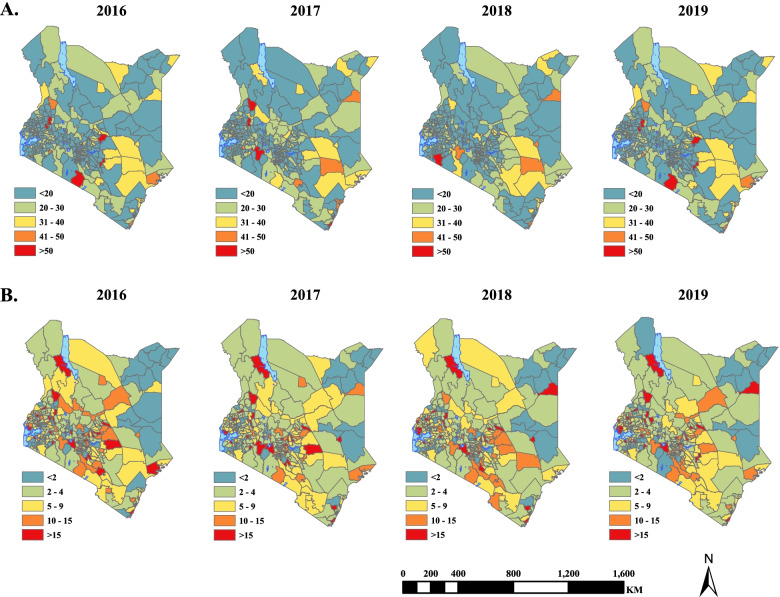
Maps showing the changing spatial evolution of the posterior median risk of (**A**) Stillbirths per 1000 live births, (**B**) Neonatal deaths per 1000 live births

The median stillbirth rate in this study was estimated to be 18.7 (95% UI: 18.0–19.4) per 1000 live births. Between 2016 to 2019, 50% or 146/290 sub-counties exhibited a decline in the risk stillbirths (ranging from an absolute difference of 20% in Kapsaret, to 0.003% in Ndhiwa), while the risk of stillbirths increased in 144 sub-counties (ranging from to 5.7% in Narok North to 0.01% in Kabondo Kasipul). Elevated risk was observed in malaria-endemic sub-counties located along the Indian ocean coastline followed by seasonal endemic sub-counties (Supplementary file 2). In 2019, the risk ranged from 87.9 (95% UI: 80.2–96.3) per 1000 live births to 4.1 (95% UI: 2.5–6.5) per 1000 live births with elevated risk observed in Narok North and Narok South sub-counties (Fig. 4).

### Neonatal deaths

The neonatal death rate was estimated to be 6.9 (6.4–7.4) per 1000 live births, with the rate declining by 2.8%, from 7.1 per 1000 live births in 2016 to 6.9 per 1000 live births in 2019. Over this period, 48% or 139/290 sub-counties exhibited a decline in the risk, with the fastest decline rate (− 2.6%) being observed in Kitutu Chache South sub-county. The risk also increased in 52% or 151/290 sub-counties, with Mvita sub-county recording the highest rise of 1.5%. Elevated risk was observed in sub-counties with urban informal settings that are characterised by poverty and poor access to maternal health services, such as Kibra and Mvita sub-counties. Similarly, malaria-endemic sub-counties along the Indian Ocean coastline and sub-counties with seasonal transmission trends exhibited elevated risk (Fig. 4).

### Impact of covariates on the proportionate rate of adverse pregnancy outcomes

The spatio-temporal distribution of covariates is shown in (Supplementary file 2). There was a positive association between maternal anaemia levels, the use of LLINs, iron supplements, folate supplements on the proportionate rate of adverse pregnancy outcomes. Conversely, our results suggested a significant negative association between adverse pregnancy outcomes and the use of IPT2 over the study period. Antenatal care was also negatively associated with adverse pregnancy outcomes, though this association was not statistically significant (Table 3).

**Table 3:** Posterior estimates of covariates

Covariate	Posterior estimate	95% Uncertainty Interval
Antenatal care – 4 visits	−0.035	−0.105, 0.100
Maternal anaemia	0.059	−0.006, 0.293
Long-lasting insecticidal-treated nets (LLINs)	0.016	−0.018, 0.073
**Intermittent preventive treatment – 2 doses (IPT2)**	**−0.071**	**−0.208, − 0.023**
Iron	0.034	−0.012, 0.145
Folate	0.084	−0.020, 0.356

**Fig. 5:**
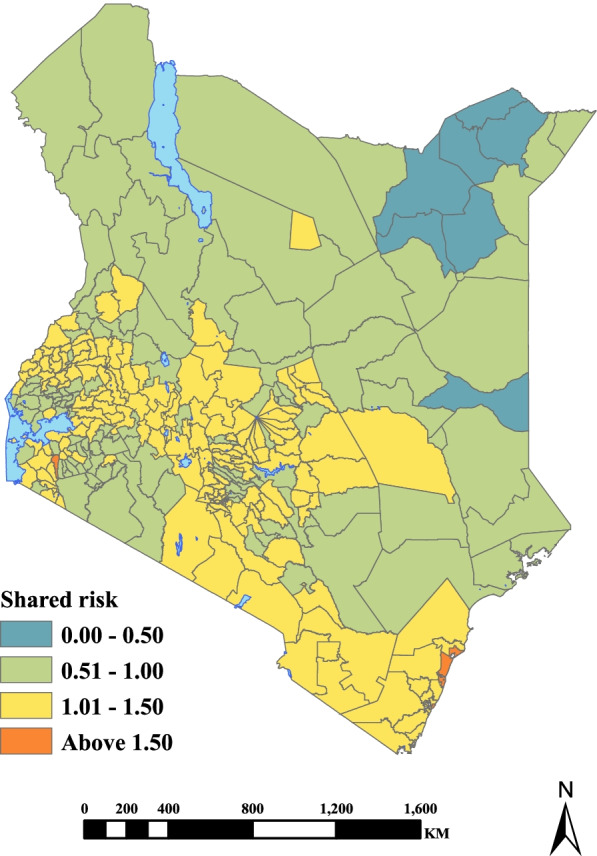
Estimated posterior median for the shared components in the model, that captures the underlying risk common to all adverse outcomes

The shared risk patterns of adverse pregnancy outcomes varied sub-nationally as displayed in Fig. 5. Shared high risk was concentrated in sub-counties located along the Indian Ocean coastline, central regions and the western parts of Kenya. These sub-counties were Nyali, Rongo, Kilifi North and Nakuru East. Conversely, the low shared risk was concentrated mainly in sub-counties located in Kenya’s northern and eastern parts such as Tarbaj, Mandera North/West, Eldas and Wajir North.

## Discussion

We employed a Bayesian hierarchical binomial model that simultaneously analysed the spatial pattern and temporal trend of adverse pregnancy outcomes (APOs) in Kenya between 2016 and 2019. Our modelling framework allowed us to incorporate information from multiple pregnancy outcomes that shared risk factors over the spatial extents and time periods. Information across the outcomes was specified via the spatial and temporal correlation structures, and our model was adjusted for both the covariate effect and an unstructured space-time interaction effect. To our knowledge, this analysis represents the first sub-national, high-resolution estimation of adverse pregnancy outcomes concomitantly over space and time in Kenya, which is a crucial input for strengthening national and county efforts towards the realisation of Universal Health Coverage (UHC) and achieving global health targets. Estimates of maternal health outcomes have historically relied on periodic household surveys such as the DHS and MIS. However, their sampling design limits their scope as they are best suited to yield estimates at the national or regional level. Compared to the unstable national-level estimates, our estimates provide greater descriptive granularity and give a baseline for bolstering maternal health programs in high-risk sub-counties.

The risk of preventable adverse pregnancy outcomes increased between 2016 and 2019 and was non-uniform across all the outcomes. Low birth weight (LBW) is considered a composite measure of foetal growth and an underlying determinant/contributor of adverse outcomes, and has been associated with an increased risk of poor physical and cognitive development in infants [41, 42]. Our results suggest an elevated risk of LBW in sub-counties located along the Indian ocean coastline which may signal the role of malaria in pregnancy. On the other hand, the low risk observed in resource-constrained sub-counties located in the north-eastern parts of Kenya may signal limited/unreliable data leading to an underestimation of its burden. This heterogeneous burden of LBW calls into question the implementation of national policies particularly in resource constrained sub-counties and urban informal settlements. Results suggest the need for more targeted approaches; particularly in sub-counties with urban informal settings where LBW contributes to neonatal deaths but remain underestimated [43, 44].

The overall pre-term birth rate was markedly lower than 9 per 100 live births previously estimated [45, 46]. By 2019, the pre-term birth rate ranged between 0.12 to 13.0 per 100 live births, with elevated estimates being observed in a few sub-counties located in the south-western parts of Kenya and along the Indian ocean coastline. However, low rates were consistently observed in the north-eastern parts of Kenya which may be attributed to limited data quantities over the period. Thus, to better understand the epidemiology of pre-term birth in low-risk sub-counties the volume and quality of routinely collected data need to be strengthened. Conversely, the elevated burden observed in some sub-counties in the south-western parts, may be reflective of the unresolved burden of pre-term births in unintended adolescent pregnancies [42, 47].

Our stillbirth estimate of 18.7 per 1000 live births was significantly lower than previous estimates for Kenya and SSA [8, 46]. The rate declined by 6.7%, from 19.4 per 1000 live births in 2016 to 18.1 per 1000 live births in 2019. The slow decline of stillbirths primarily attributed to intrapartum deaths [8], may be reflective on the inadequate global and national attention in mitigating the burden [11]. Additionally, generated estimates need to be interpreted cautiously due to under-reporting attributed to recall bias, inconsistent definitions/misclassification of fresh versus macerated stillbirths and omission. This may limit comparison with other international studies using diverse classification criteria [7, 11]. Improving the quality of care and birth and registry data will likely contribute to accurate estimates sub-nationally.

Neonatal death rate is an important index for tracking progress towards the attainment of SDG targets, that seeks to reduce neonatal mortality to at least 12 deaths per 1000 live births. Understanding its spatial extents and temporal trends is essential/critical due to its rising share in under-five mortality in Kenya [25]. Our estimates was markedly lower compared to a facility-based study that estimated the neonatal death rate to be 16 per 1000 live births [46]. Huge disparities in neonatal deaths persisted across sub-counties, with elevated risk observed in malaria-endemic regions located along the Indian Ocean coastline and Lake Victoria region. The highest risk was consistently observed in Kibra sub-county, with approximately 75 deaths in 1000 live births [48]. It is important to note that Kibra inhabits the largest urban informal settlement in Africa [44]. Thus, the elevated risk may have been compounded by poor sanitation practices and over-stretched facilities in the densely populated informal settlements [49].

Our results suggest that disparities in the spatial extents of APOs may have been driven by demand-side barriers and supply-side challenges, with low-resource counties disproportionately vulnerable to adverse outcomes. Recent studies highlight a heterogenous timing and coverage of ANC4 across sub-counties in Kenya, with poorer/rural sub-counties in northern Kenya exhibiting low coverage levels [50, 51]. The current guidance from the WHO suggesting at least four comprehensive and targeted visits [16, 52]. However, a confidential inquiry into maternal deaths in Kenya indicated that only one in five women attending ANC had at least 4 ANC visits in 2014 [53]. ANC attendance and coverage are essential as they are positively associated with increased use of birth attendants, reducing the risk of adverse pregnancy outcomes. Pregnant women are screened for anaemia, HIV, syphilis, urinalysis, and blood typing (ANC profile) during these visits. Additionally, they receive vital micronutrient supplementation essential to both the mother and her unborn child among other interventions such as educational information on safe motherhood. However, regional disparities may signify the inequitable access and the widespread unmet need of ANC services particularly in arid, resource-constrained, rural and pastoralist dominated sub-counties located eastern and northern parts of Kenya as previously documented [11, 54–56].

Increasing concerns about the shortage and distribution of skilled healthcare workers sub nationally, adequacy of pre-existing healthcare infrastructure, stakeholder non-involvement in policy formulation has previously been raised [10, 57]. These concerns have been exacerbated by the distance to health facility, timely access to quality maternal care, cost of transport, poverty, illiteracy, religious and social-cultural beliefs and tendencies. These factors tend to suppress the uptake of facility-based delivery with only the complicated cases being referred to the facility [50, 51, 58–61]. This is more pronounced in the rural and pastoralist communities that prefer traditional birth attendants [62, 63]. According to the Kenya Health Workforce Status Report in 2017, the nurse-to-population ratio of 0.1/10,000 in arid and semi-arid sub-counties in northern Kenya was markedly lower than 9.7/10,000 in sub-counties Nairobi [64]. This might have contributed by high attrition rates of health staff in these sub-counties due to poor infrastructure and insecurity. This has led to poor utilisation of public health facilities, leading to increased home deliveries [61]. Our results lend credence to efforts to strengthen the healthcare workforce through policy, planning, adequate financing, recruitment, training and deployment. This may help to improve sub-county health systems and help achieve global and national health targets such as the universal coverage of skilled birth attendants [65].

In low-resource sub-counties in northern Kenya, macronutrient/maternal undernutrition - caused by inadequate intake of essential minerals and vitamins; contributes to anaemia in pregnancy, which might also precipitate APOs [66]. Additionally, infectious diseases such as malaria and human immunodeficiency virus (HIV) have previously been associated with an increased risk of pre-term deliveries, stillbirths, and neonatal deaths. The burden is more pronounced in sub-counties located in western Kenya [67–73]. Sexually transmitted infections such as chlamydia, gonorrhoea, trichomoniasis and syphilis have also been associated with an increased risk of stillbirths and low birth weight among pregnant women in rural settings [74–76].

### Implication of study

Despite the achievements in maternal and child health-related programs over the recent decades, preventable adverse pregnancy outcomes remain a key challenge to pregnant women and their infants. The study presents a unique dimension for prioritising high-risk areas and increasing the efficiency of control programmes to alleviate the burden imposed by preventable APOs. This will accelerate the attainment of maternal health targets and align sub-county estimates with global and national priorities. Crucially, our estimates can help to raise awareness of growing inequities in Kenya’s diverse and growing population inhabiting different regions with diverse levels of social and economic development.

The shared risk estimates underscore the need to establish integrated suites of interventions (policy/care package/services) at meaningful policy levels to reduce missed opportunities/comprehensively address healthcare needs of pregnant women at the sub-county level, especially through nutrition-specific and nutrition-sensitive interventions. Compared to the national level estimates, our joint estimates resonate with Kenya’s broader maternal health objectives, focusing on disease patterns and monitoring/accelerating progress in maternal health care equity as outlined in the SDGs. Our findings also inform the need of mapping the overlapping burden of infectious diseases and APOs in Kenya. Study approaches can also be applied to other health outcomes with shared risk factors.

As more data on maternal health outcomes becomes available, underestimation stemming from inaccurate reporting/documentation can be used to flag sub-counties with less reliable data on other maternal health indicators and help channel investments to sub-counties with poor surveillance systems, constrained diagnostic capacity and unmet reproductive health needs. Overall, the local burden of disease can be used to prioritise appropriate, cost-effective interventions targeting the poor, rural sub-counties and informal settlements in urban areas will likely address the wide disparities and accelerate progress in maternal and newborn health. There is also a pressing need to understand more about APO’s risk to urban slum dwellers in Kenya who are disproportionately bombarded with chronic illness, maternal illiteracy, and socio-economic instability.

### Study strengths and limitations

At a much higher level of spatio-temporal precision, we demonstrate the importance of a functional health information system in providing routine estimates between periodic and expensive survey estimates. Facility-based data can help inform the local burden of disease. The continual adoption of standard definitions and consistent measurement procedures will be critical for meaningful comparisons towards the attainments of SDGs. The Bayesian shared component model adds versatility to substantive epidemiological questions. Its ability to borrow strength from outcomes and across neighbouring sub-counties makes it an important tool for monitoring the evolution of facility-based maternal health disparities across the continuum of care.

Our findings need to be interpreted cautiously due to several methodological challenges that might have confounded our estimates. First, the study presents facility-based estimates that may not be generalisable to the entire population, especially for women delivering outside the public health facilities. This may be due to the geographic underrepresentation of public antenatal clinics. Secondly, we cannot discount the potential incompleteness of our data, disproportionately available in malaria endemic sub-counties with more robust surveillance infrastructure and higher uptake of antenatal care services. Third, misclassification of pregnancy outcomes; e.g. neonatal deaths as stillbirths, pre-term birth as low birth weight (or vice versa), might have led to underestimating some adverse outcomes. This may in part explain previous concerns raised on the use of routine health metrics for international comparisons [77]. Fourth, although we included covariates to stabilise our estimates, our study was ecological with data aggregated at the sub-county and yearly level; this might have led to residual confounding. Fifth, our study did not account for the adverse outcomes during the different pregnancy semesters, pregnancy history, underlying conditions and parity which has previously been associated with adverse outcomes.

## Conclusions

Our study primarily modelled the geographical distribution and risk of adverse pregnancy outcomes among women presenting at public health facilities in Kenya. High-resolution estimates via the Bayesian joint spatio-temporal models were used to strengthen inference by borrowing information across adjacent regions and adverse pregnancy outcomes with common risk factors. As facility-based deliveries continue to increase, there is an urgent need to improve the quality and volume of data, including standardising definitions, measurements and reporting. This will enable the implementation of appropriate context-specific interventions and help reduce associated APOs. Over the coming decades, understanding the joint burden of overlooked maternal health disparities indexed in space and time will enable the implementation of health measures with increasing granularity, simultaneously decreasing the risk of adverse pregnancy outcomes.

## Supplementary Information

**Additional file 1: Supplementary file 1**: Additional modelling details

**Additional file 2: Supplementary file 2**: Additional data descriptions, methodological information and results

## Data Availability

The datasets analysed during the current study are available in the Kenya District Health Information Software online database (DHIS2) repository, http://www.hiskenya.org. The shapefile used in the study was downloaded from https://data.humdata.org/dataset/ken-administrative-boundaries.

## Abbreviations

CAR: Conditional autoregressive
DIC: Deviance information criterion
MCMC: Markov chain Monte Carlo
DHIS: District health information system
SSA: Sub-Saharan Africa
SDG: Sustainable development goal
WHO: World health organization
ENAP: Every newborn action plan
EPMM: Ending preventable maternal mortality
