# Supplementary material for: Joint spatio-temporal modelling of adverse pregnancy outcomes sharing common risk factors at sub-county level in Kenya, 2016–2019
Source: BMC Public Health. 2021 Dec 30;21:2331. doi: 10.1186/s12889-021-12210-9 (PMC8719408; doi:10.1186/s12889-021-12210-9)
Supplement: Supplementary file 1 — Additional file 1: Supplementary file 1: Additional modelling details [file 12889_2021_12210_MOESM1_ESM.docx]

**Supplementary material: Methodological information and results**

**S1: Joint Spatio-temporal model**

***Hierarchical joint spatio-temporal model structure***

The joint spatio-temporal model structure consists of three levels namely; the data level, process level and the parameter levels. The data level exhibits an independent conditional probability distribution subject to the process and modelling parameters whereas the process level determines the changes within the data level subject to the parameters.

**Data level**

Let $\boldsymbol{Y}_{\boldsymbol{ijk}}$ be the observed number of adverse pregnancy outcomes, where $\boldsymbol{i}$ represents a given sub-county (1 – 290), $\boldsymbol{j}$ represents different time periods (2016 – 2019) and $\boldsymbol{k}$ represents the different adverse pregnancy outcomes (*1 – LBW, 2 – Preterm births, 3 – Still births, 4 – Neonatal deaths*).

Let $\boldsymbol{n}_{\boldsymbol{ijk}}$ be total live births for sub-county $\boldsymbol{i (i=1,\ldots,290)}$ and time period $\boldsymbol{j (j=1,\ldots,4)}$**.**

Due to the low rate of adverse pregnancy outcomes when compared to livebirths; and without the loss of generality a binomial model for observed counts with a logit link function was used in the analysis.

i.e. We assume that the observed number of cases $\boldsymbol{Y}_{\boldsymbol{ijk}}$ arises from a binomial distribution, that is;

$\boldsymbol{Y}_{\boldsymbol{ijk}}\boldsymbol{\sim binomial(}\boldsymbol{n}_{\boldsymbol{ijk}}\boldsymbol{,\pi}_{\boldsymbol{ijk}}\boldsymbol{)}$ $\boldsymbol{i=1,\ldots, 290; j=2016,\ldots,2019; k=1,2,3,4}$

Here, $\pi_{ijk}$ represents the true, unknown risk proportion in sub-county $\boldsymbol{i}$**.** time $\boldsymbol{j}$ and outcome $\boldsymbol{k.}$

**Process model**

Here we propose a latent spatial to represent relationships between adjacent sub-counties and a latent temporal process characterise correlation over time respectively. The spatio-temporal relationship is driven by common risk factors.

Then the risk rate is specified on the logit scale as:

$$Logit\left( \boldsymbol{\pi}_{\boldsymbol{ijk}} \right)= \alpha_{k}+\boldsymbol{X}_{\boldsymbol{i}}\beta_{k}+\boldsymbol{X}_{\boldsymbol{ij}}\beta_{k}+ \boldsymbol{\mu}_{\boldsymbol{ijk}}$$

The parameter $\alpha_{k}$ is the adverse pregnancy outcome specific intercepts, $\boldsymbol{X}_{\boldsymbol{i}}$ and $\boldsymbol{X}_{\boldsymbol{ij}}$ are the time in-invariant spatial covariates and space-time varying covariates respectively. Whereas $\beta_{k}$ are the vectors of regression coefficients for each pregnancy outcome that accounts for the varied risk gradients of the shared spatial and temporal components. i.e. they represent the relative weight of the contribution of the shared terms to the risk APOs. The spatio-temporal structure is represented by $\boldsymbol{\mu}_{\boldsymbol{ijk}}$**,** which accounts for the possible variations in the logit scale.

Then the joint spatial-temporal structure for each outcome was specified as follows^1-3^

$$\boldsymbol{\mu}_{\boldsymbol{ij}\boldsymbol{k}}\boldsymbol{=}\boldsymbol{\gamma}_{\boldsymbol{k}}^{\boldsymbol{s}}\boldsymbol{u}_{\boldsymbol{i}}^{\boldsymbol{s}}\boldsymbol{+}\boldsymbol{u}_{\boldsymbol{i}\boldsymbol{k}}^{\boldsymbol{s}}\mathbf{+}\boldsymbol{\gamma}_{\boldsymbol{k}}^{\boldsymbol{t}}\boldsymbol{u}_{\boldsymbol{j}}^{\boldsymbol{t}}\mathbf{+}\boldsymbol{u}_{\boldsymbol{j}\boldsymbol{k}}^{\boldsymbol{t}}\mathbf{+}\boldsymbol{\nu}_{\boldsymbol{ij}}$$

With $\sum_{k=1}^{4} \gamma_{k}^{s}=0$, $\sum_{k=1}^{4} {log \gamma}_{k}^{t}=0$

In this formulation $u_{i}^{s}$ are a set of common random effects associated with space (CAR), $u_{ik}^{s}$ are outcome-specific random effects associated with space (CAR), $k=1,2,3,4$,$u_{j}^{t}$ are a set of common random effects associated with time (RW1), $u_{jk}^{t}$ are outcome-specific random effects associated with time (RW1). The space-time interaction term/heterogeneity of order two is represented by $\boldsymbol{\nu}_{\boldsymbol{ij}}$ i.e. it represents the possible variations not explained by the spatial and temporal effects in the model.

**Parameter model**

- ***Spatial random effects***

To capture the local dependence in space, the random spatial effects $\boldsymbol{\lambda}$ were modelled using a conditional autoregressive (CAR) Gaussian distribution. It implied that the conditional distribution of each $\boldsymbol{\lambda}_{\boldsymbol{i}}$ given all $\boldsymbol{\lambda's}$ is normally distributed with mean equal to the average of the $\boldsymbol{\lambda's}$ of its neighbouring polygons (sub-counties), and precision proportional to the number of ‘neighbours’. i.e. $\boldsymbol{\lambda}^{\boldsymbol{'}}\boldsymbol{s\sim}CARNormal\boldsymbol{(W,\tau\lambda)}$**.**

A first order queen contingency (i.e. all sub-counties which share a common boundary) neighbourhood structure was utilized in the CAR via a 290 x 290 adjacency/neighbourhood matrix $\boldsymbol{W=}\boldsymbol{(W}_{\boldsymbol{uv}}\boldsymbol{)}$ where $\boldsymbol{W}_{\boldsymbol{uv}}\boldsymbol{=1}$ if two sub-counties say $u and v$ share a common boundary and 0 otherwise $\boldsymbol{W}_{\boldsymbol{uu}}$

- ***Temporal random effects***

We assumed a first order (pre and post) random RW (1) to define the temporal effects$(\boldsymbol{\xi)}$ across different time periods, or one-dimensional versions of the CAR spatial priors, with the weight matrix $\boldsymbol{Q}$ defining the temporal neighbourhood structure $j$as years $j-1$ and $j+1$ from 2016 – 2019. i.e. $\boldsymbol{\xi}^{\boldsymbol{'}}\boldsymbol{s\sim}CAR.Normal\boldsymbol{(Q,\tau\xi)}$**.**

**Model specification**

The scaling parameters $\boldsymbol{\delta}and \boldsymbol{\kappa}$ represent the relative contribution of the shared terms to the risk of given outcome to the overall APOs spatial and temporal effects respectively. To ensure model identifiability^4^, we implemented a sum to zero constraint for $\boldsymbol{\delta}_{\boldsymbol{1-4}}$ and $\boldsymbol{\kappa}_{\boldsymbol{1-4}}$**.**

We assumed an exchangeable (unstructured) hierarchical structure for the shared interaction terms $\boldsymbol{\nu}_{\boldsymbol{ij}}$**.** i.e. $\boldsymbol{\nu}_{\boldsymbol{ij}}\boldsymbol{\sim}Normal\boldsymbol{(0,\tau\nu)}$**.**

For the regression coefficients $\beta_{k}$, we assumed they followed a non-informative normal prior distribution.

Based on previous studies, the precision parameters of the spatial and temporal CAR priors were assigned a weak informative independent hyper-prior Gamma distribution. i.e. $\boldsymbol{\tau}_{\boldsymbol{s}}\boldsymbol{\sim}Gamma\boldsymbol{(}0.5,0.0005\boldsymbol{)}$^5^**.** We also assigned multivariate normal distribution for both the logarithm of the spatial and temporal scale parameter, $\boldsymbol{log \delta, log \kappa\sim}Normal\boldsymbol{(}0.5,0.0005\boldsymbol{)}$.

**S2: WinBUGS code for model implementation**

Model

{

b.x3~dflat()

b.x4~dflat()

b.x5~dflat()

b.x6~dflat()

for (i in 1:N) {

for (j in 1:T) {

#Binomial likelihood for observed counts

lbw[i,j]~dbin(p1[i,j],lb[i,j])

ptb[i,j]~dbin(p2[i,j],lb[i,j])

sb[i,j]~dbin(p3[i,j],lb[i,j])

nd[i,j]~dbin(p4[i,j],lb[i,j])

#Scaling of covariates

xs1[i,j]<-x1[i,j]/anc1[i]

xs2[i,j]<-x2[i,j]/anc1[i]

x3[i,j]~dpois(mu.x3[i,j])

log(mu.x3[i,j])<-log(anc1[i])+b.x3+phi.x3[i]+gamma.x3[j]

xs3[i,j]<-mu.x3[i,j]/anc1[i]

x4[i,j]~dpois(mu.x4[i,j])

log(mu.x4[i,j])<-log(anc1[i])+b.x4+phi.x4[i]+gamma.x4[j]

xs4[i,j]<-mu.x4[i,j]/anc1[i]

x5[i,j]~dpois(mu.x5[i,j])

log(mu.x5[i,j])<-log(anc1[i])+b.x5+phi.x5[i]+gamma.x5[j]

xs5[i,j]<-mu.x5[i,j]/anc1[i]

x6[i,j]~dpois(mu.x6[i,j])

log(mu.x6[i,j])<-log(anc1[i])+b.x6+phi.x6[i]+gamma.x6[j]

xs6[i,j]<-mu.x6[i,j]/anc1[i]

#Risk factor model with covariates

logit(p1[i,j])<-alpha[1]+s[1]*b[1]*xs1[i,j]+s[2]*b[2]*xs2[i,j]+s[3]*b[3]*xs3[i,j]+s[4]*b[4]*xs4[i,j]+s[5]*b[5]*xs5[i,j]+mu[i,j,1]

logit(p2[i,j])<-alpha[2]+s[6]*b[1]*xs1[i,j]+s[7]*b[2]*xs2[i,j]+s[8]*b[3]*xs3[i,j]+mu[i,j,2]

logit(p3[i,j])<-alpha[3]+s[9]*b[1]*xs1[i,j]+s[10]*b[2]*xs2[i,j]+s[11]*b[3]*xs3[i,j]+s[12]*b[4]*xs4[i,j]+mu[i,j,3]

logit(p4[i,j])<-alpha[4]+s[13]*b[1]*xs1[i,j]+s[14]*b[2]*xs2[i,j]+s[15]*b[4]*xs4[i,j]+s[16]*b[6]*xs6[i,j]+mu[i,j,4]

mu[i,j,1:4]~dmnorm(eta[i,j,],Sigma.inv[,])

#Joint modelling

eta[i,j,1]<-delta[1]*phi[i]+gamma[j]*kappa[1]+epsilon[i,1]

eta[i,j,2]<-delta[2]*phi[i]+gamma[j]*kappa[2]+epsilon[i,2]

eta[i,j,3]<-delta[3]*phi[i]+gamma[j]*kappa[3]+epsilon[i,3]

eta[i,j,4]<-delta[4]*phi[i]+gamma[j]*kappa[4]+epsilon[i,4]

}}

for (i in 1:N) {

epsilon[i,1:4] ~ dmnorm(mean[1:4], P[1:4, 1:4])

}

for (j in 1:4) {

mean[j] <-0.0

}

P[1:4,1:4] ~ dwish(Q[1:4, 1:4],4)

for (i in 1:4) {

for (j in 1:4) {

Q[i,j]<-0.01*equals(i,j)}}

#Spatial Modelling (priors)

phi[1:N]~car.normal(adj[],weights[],num[],tau.phi[1])

phi.x3[1:N]~car.normal(adj[],weights[],num[],tau.phi[2])

phi.x4[1:N]~car.normal(adj[],weights[],num[],tau.phi[3])

phi.x5[1:N]~car.normal(adj[],weights[],num[],tau.phi[4])

phi.x6[1:N]~car.normal(adj[],weights[],num[],tau.phi[5])

#Weights for adjacency matrices in space

for(k in 1:sumNumNeigh) {

weights[k]<-1

}

#Temporal Modelling (priors)

gamma[1:T]~car.normal(adj.t[],weights.t[],num.t[],tau.gamma[1])

gamma.x3[1:T]~car.normal(adj.t[],weights.t[],num.t[],tau.gamma[2])

gamma.x4[1:T]~car.normal(adj.t[],weights.t[],num.t[],tau.gamma[3])

gamma.x5[1:T]~car.normal(adj.t[],weights.t[],num.t[],tau.gamma[4])

gamma.x6[1:T]~car.normal(adj.t[],weights.t[],num.t[],tau.gamma[5])

for(t in 1:1){

weights.t[t] <- 1;

adj.t[t] <- t+1;

num.t[t] <- 1

}

for(t in 2:(T-1)) {

weights.t[2+(t-2)*2] <- 1;

adj.t[2+(t-2)*2] <- t-1

weights.t[3+(t-2)*2] <- 1;

adj.t[3+(t-2)*2] <- t+1;

num.t[t] <- 2

}

for(t in T:T) {

weights.t[(T-2)*2 + 2] <- 1;

adj.t[(T-2)*2 + 2] <- t-1;

num.t[t] <- 1

}

#Hyperprior specification

for(k in 1:5) {

tau.phi[k]~dgamma(0.5, 0.005)

}

for(k in 1:5) {

tau.gamma[k]~dgamma(0.5, 0.005)

}

for (j in 1:4) {

logdelta[j] ~ dnorm(0, 5.9)

delta[j] <- exp(logdelta[j])

logkappa[j] ~ dnorm(0, 5.9)

kappa[j] <- exp(logkappa[j])

}

#Intercepts

for (k in 1:4){

alpha[k]~dnorm(0,tau.alpha[k])

tau.alpha[k]~dgamma(2,0.5)

}

for(k in 1:4) {

tau0[k]~dgamma(2,0.5)

}

Sigma.inv[1:4,1:4]~dwish(B[,],4)

B[1,1]<-0.01

B[2,2]<-0.01

B[3,3]<-0.01

B[4,4]<-0.01

B[1,2]<-0

B[1,3]<-0

B[1,4]<-0

B[2,1]<-0

B[2,3]<-0

B[2,4]<-0

B[3,1]<-0

B[3,2]<-0

B[3,4]<-0

B[4,1]<-0

B[4,2]<-0

B[4,3]<-0

#Coefficient for covariates

b[1]~dnorm(0,0.01)

b[2]~dnorm(0,0.01)

b[3]~dnorm(0,0.01)

b[4]~dnorm(0,0.01)

b[5]~dnorm(0,0.01)

b[6]~dnorm(0,0.01)

for(k in 1:16) {

s[k]~dunif(0.4,2.5)

}

# relative risks and other summary quantities

for (i in 1 : N) {

sharedRR[i]<- exp(phi[i]) # overall RR of shared in subcounty i

prob.sharedRR[i]<-step(sharedRR[i]-1)

}

for (i in 1 : N) {

for (j in 1:4) {

especificRR[i,j]<-exp(epsilon[i,j]) # residual RR of dis[j] in subcounty i not explained by shared component

}

}

for (j in 1:4) {

var.especific[j]<-sd(epsilon[,j])*sd(epsilon[,j]) #empirical variance of disease specific effects

var.shared[j]<-pow(delta[j],2)*sd(phi[])*sd(phi[]) #empirical variance of shared components

frac.shared[j] <-(pow(delta[j],2)*sd(phi[])*sd(phi[])) / (var.shared[j] + (sd(epsilon[,j])*sd(epsilon[,j])))

}

}

}

**S3: Model Convergence**

**Gelman-Rubin convergence plots for key model parameters**

To ensure the reliability of the posterior estimates, convergence was assessed by visual inspection of the parameter series plot/trace plots and analytically by the Gelman-Rubin statistics^6^. The posterior estimates (fixed effects, scaling parameters, and standard deviations of all random effects) were also assessed to check if the MCMC simulation had converged. i.e. A MC error/SD of less than 5% pointed to the model stability/convergence.

**Table 1: Parameter posterior distribution­ with Monte Carlo (MC) error divided by the standard deviation (SD)**

| **node** | **mean** | **SD** | **MC error** | **2.50%** | **median** | **97.50%** | **start** | **sample** | **MC error/SD (%)** |
| --- | --- | --- | --- | --- | --- | --- | --- | --- | --- |
| alpha[1] | -3.247 | 0.2987 | 0.01446 | -3.373 | -3.21 | -3.14 | 4001 | 76000 | 4.840978 |
| alpha[2] | -4.112 | 0.1799 | 0.008983 | -4.24 | -4.129 | -4.022 | 4001 | 76000 | 4.99333 |
| alpha[3] | -4.074 | 0.08415 | 0.004135 | -4.172 | -4.08 | -3.986 | 4001 | 76000 | 4.913844 |
| alpha[4] | -5.413 | 0.2093 | 0.01038 | -5.573 | -5.432 | -5.284 | 4001 | 76000 | 4.959388 |
| b[1] | -0.02371 | 0.09049 | 0.003669 | -0.1046 | -0.03544 | 0.09965 | 4001 | 76000 | 4.054592 |
| b[2] | 0.07441 | 0.07293 | 0.00346 | -0.00627 | 0.05866 | 0.2932 | 4001 | 76000 | 4.744275 |
| b[3] | 0.01879 | 0.0233 | 0.00113 | -0.01772 | 0.01565 | 0.07334 | 4001 | 76000 | 4.849785 |
| b[4] | -0.08164 | 0.0454 | 0.002138 | -0.2078 | -0.07141 | -0.0225 | 4001 | 76000 | 4.709251 |
| b[5] | 0.04318 | 0.0398 | 0.001684 | -0.01154 | 0.03394 | 0.1446 | 4001 | 76000 | 4.231156 |
| b[6] | 0.1054 | 0.09317 | 0.003942 | -0.01966 | 0.08354 | 0.3561 | 4001 | 76000 | 4.230976 |
| delta[1] | 1.728 | 0.3998 | 0.0186 | 1.019 | 1.697 | 2.606 | 4001 | 76000 | 4.652326 |
| delta[2] | 2.017 | 0.468 | 0.02157 | 1.197 | 1.991 | 3.015 | 4001 | 76000 | 4.608974 |
| delta[3] | 0.2951 | 0.08203 | 0.00265 | 0.163 | 0.2853 | 0.4812 | 4001 | 76000 | 3.230525 |
| delta[4] | 1.273 | 0.3143 | 0.01396 | 0.7476 | 1.24 | 1.969 | 4001 | 76000 | 4.441616 |
| kappa[1] | 2.464 | 0.859 | 0.02347 | 0.5106 | 2.387 | 4.34 | 4001 | 76000 | 2.732247 |
| kappa[2] | 0.8017 | 0.3035 | 0.00647 | 0.3711 | 0.7513 | 1.537 | 4001 | 76000 | 2.131796 |
| kappa[3] | 0.5172 | 0.2157 | 0.006645 | 0.2467 | 0.4763 | 1.077 | 4001 | 76000 | 3.080668 |
| kappa[4] | 0.8223 | 0.2928 | 0.003969 | 0.3841 | 0.7787 | 1.518 | 4001 | 76000 | 1.355533 |

**Gelman – Rubin diagnostic check**

**Figure 2: Model convergence:** Gelman-Rubin Brooks diagnostics plots demonstrating convergence during the MCMC simulation for key model parameters. As inspection of these plots, suggest convergence/stabilization of model after approximately 80000 iterations

|                     |
| --- |

**S4: Model validation:**

**Scatter plots and correlation coefficients for observed rates versus model posterior rates**


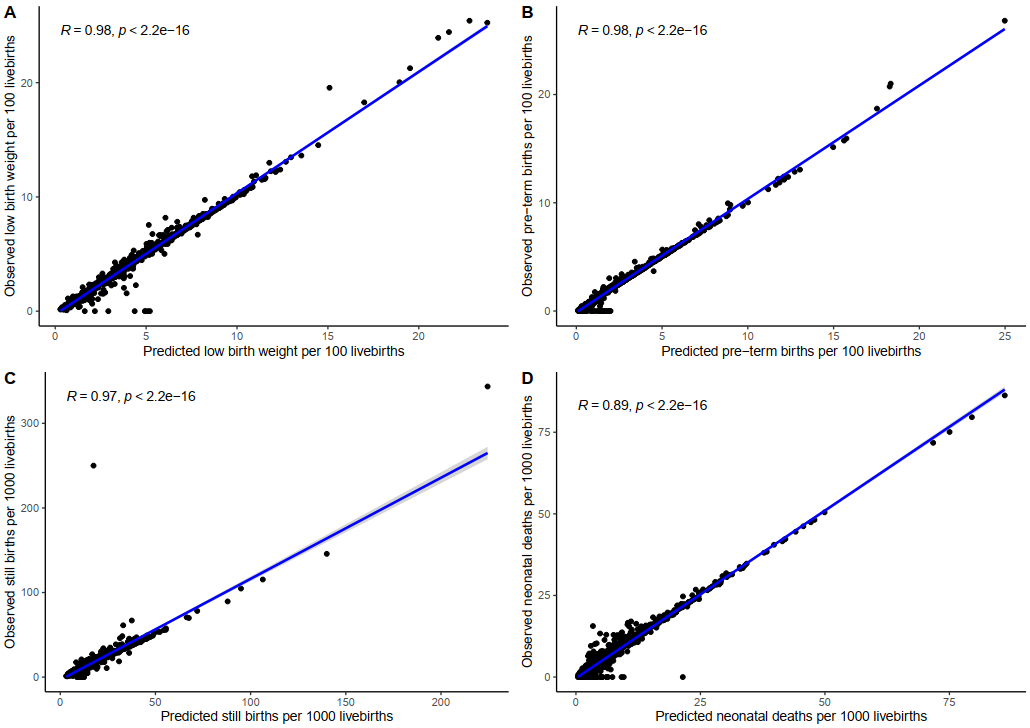


**Figure 3: A comparison between the observed rates versus fitted posterior rates**. **(A):** Low birth weight: spearman rho = +0.98, P<0.001; **(B):** pre-term birth spearman rho = +0.98, P<0.001; **(C):** still births: spearman rho = +0.97, P<0.001; **(D):** neonatal deaths: spearman rho = +0.995, P<0.001).

Our joint model suggested a very high degree of correlation. with a few notable differences e.g. many sub-counties with missing counts or observed rates of zero were corrected upwards by the model and/or smoothed towards local areal mean based on contiguity matrix in space and time (Figure 1).

**S5: Out of sample validation**

A random 20% of observed data points was drawn from the space-time cube. The data with the removed points were then re-inputted into WinBUGS. To ascertain the predictive power of the model the posterior distribution of the data points was then compared with the observed values. 86% (199/232) of the observed values were within the 95% credible interval in the posterior distribution. The correlation coefficient was +0.85, P<0.001 (Figure 2).


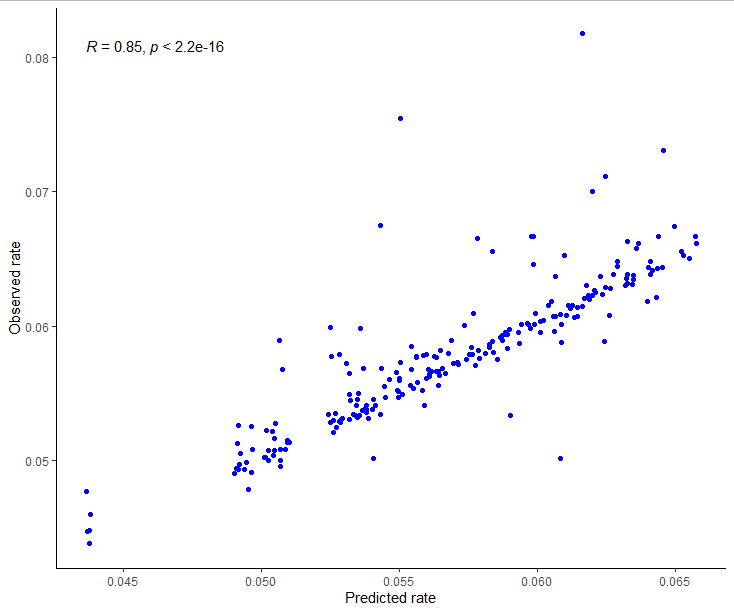


**Figure 4:** Out of sample validation between the observed and predicted proportionate rate

**List of references**

1. Knorr‐Held L. Bayesian modelling of inseparable space‐time variation in disease risk. *Statistics in medicine* 2000;19(17‐18):2555-67.

2. Richardson S, Abellan JJ, Best N. Bayesian spatio-temporal analysis of joint patterns of male and female lung cancer risks in Yorkshire (UK). *Statistical methods in medical research* 2006;15(4):385-407.

3. Knorr‐Held L, Best NG. A shared component model for detecting joint and selective clustering of two diseases. *Journal of the Royal Statistical Society: Series A (Statistics in Society)* 2001;164(1):73-85.

4. MacNab YC. On Bayesian shared component disease mapping and ecological regression with errors in covariates. *Statistics in medicine* 2010;29(11):1239-49.

5. Wakefield J, Best N, Waller L. Bayesian approaches to disease mapping. *Spatial epidemiology: methods and applications* 2000:104-27.

6. Gelman A, Rubin DB. Inference from iterative simulation using multiple sequences. *Statistical science* 1992;7(4):457-72.
