# Supplementary material for: Joint spatio-temporal modelling of adverse pregnancy outcomes sharing common risk factors at sub-county level in Kenya, 2016–2019
Source: BMC Public Health. 2021 Dec 30;21:2331. doi: 10.1186/s12889-021-12210-9 (PMC8719408; doi:10.1186/s12889-021-12210-9)
Supplement: Supplementary file 2 — Additional file 2: Supplementary file 2: Additional data descriptions, methodological information and results [file 12889_2021_12210_MOESM2_ESM.docx]

**Supplementary Material: Additional data descriptions, methodological information and results**

**Table 1: List of sub-counties (numbered) and their corresponding county and malaria endemicity as presented in Figure 1**

| **Endemicity** | **County** | **Sub-county** |
| --- | --- | --- |
| **Coast endemic** | Mombasa, Kwale, Kilifi, Taita Taveta | Changamwe [1], Jomvu[2], Kisauni[3], Nyali[4], Likoni[5], Mvita[6], Msambweni[7], Lunga Lunga[8], Matuga[9], Kinango[10], Kilifi North[11], Kilifi South[12], Kaloleni[13], Rabai[14], Ganze[15], Malindi[16], Magarini[17], Lamu East[21], Lamu West[22], Taveta[23], Wundanyi[24], Mwatate[25], Voi[26]. |
| **Highland** | West Pokot, Trans Nzoia, Uasin Gishu, Nandi, Baringo, Narok, Kericho, Bomet, Kisii, Nyamira | Kapenguria[129], Sigor[130], Kacheliba[131], Pokot South[132], Kwanza[136], Endebess[137], Saboti[138], Kiminini[139], Cherangany[140], Soy[141], Turbo[142], Moiben[143], Ainabkoi[144], Kapseret[145], Kesses[146], Tinderet[151], Aldai[152], Nandi Hills[153], Chesumei[154], Emgwen[155], Mosop[156], Tiaty[157], Baringo North[158], Baringo Central[159], Baringo South[160], Mogotio[161], Eldama Ravine[162], Kilgoris[177], Emurua Dikirr[178], Narok West[179], Narok North[180], Narok East[181], Narok South[182], Kipkelion East[188], Kipkelion West[189], Ainamoi[190], Bureti[191], Belgut[192], Sigowet/Soin[193], Sotik[194], Chepalungu[195], Bomet East[196], Bomet Central[197], Konoin[198], Bonchari[261], South Mugirango[262], Bomachoge Borabu[263], Bobasi[264], Bomachoge Chache[265], Nyaribari Masaba[266], Nyaribari Chache[267], Kitutu Chache North[268], Kitutu Chache South[269], Kitutu Masaba[270], West Mugirango[271], North Mugirango[272], Borabu[273]. |
| **Lake endemic** | Kakamega, Vihiga, Bungoma, Busia, Siaya, Kisumu, Homa Bay, Migori | Lugari[199], Likuyani[200], Malava[201], Lurambi[202], Navakholo[203], Mumias West[204], Mumias East[205], Matungu[206], Butere[207], Khwisero[208], Shinyalu[209], Ikolomani[210], Vihiga[211], Sabatia[212], Hamisi[213], Luanda[214], Emuhaya[215], Mt. Elgon[216], Sirisia[217], Kabuchai[218], Bumula[219], Kanduyi[220], Webuye East[221], Webuye West[222], Kimilili[223], Tongaren[224], Teso North[225], Teso South[226], Nambale[227], Matayos[228], Butula[229], Funyula[230], Budalangi[231], Ugenya[232], Ugunja[233], Alego Usonga[234], Gem[235], Bondo[236], Rarieda[237], Kisumu East[238], Kisumu West[239], Kisumu Central[240], Seme[241], Nyando[242], Muhoroni[243], Nyakach[244], Kasipul[245], Kabondo Kasipul[246], Karachuonyo[247], Rangwe[248], Homa Bay Town[249], Ndhiwa[250], Suba North[251], Suba South[252], Rongo[253], Awendo[254], Suna East[255], Suna West[256], Uriri[257], Nyatike[258], Kuria West[259], Kuria East[260]. |
| **Low risk** | Machakos, Makueni, Nyandarua, Nyeri, Kirinyaga, Muranga, Kiambu, Laikipia, Nakuru, Nairobi | Masinga[75], Yatta[76], Kangundo[77], Matungulu[78], Kathiani[79], Mavoko[80], Machakos Town[81], Mwala[82], Mbooni[83], Kilome[84], Kaiti[85], Makueni[86], Kibwezi West[87], Kibwezi East[88], Kinangop[89], Kipipiri[90], Olkalou[91], Ol Jorok[92], Ndaragwa[93], Tetu[94], Kieni[95], Mathira[96], Othaya[97], Mukurweini[98], Nyeri Town[99], Mwea[100], Gichugu[101], Ndia[102], Kirinyaga Central[103], Kangema[104], Mathioya[105], Kiharu[106], Kigumo[107], Maragwa[108], Kandara[109], Gatanga[110], Gatundu South[111], Gatundu North[112], Juja[113], Thika Town[114], Ruiru[115], Githunguri[116], Kiambu Town[117], Kiambaa[118], Kabete[119], Kikuyu[120], Limuru[121], Lari[122], Laikipia West[163], Laikipia East[164], Laikipia North[165], Molo[166], Njoro[167], Naivasha[168], Gilgil[169], Kuresoi South[170], Kuresoi North[171], Subukia[172], Rongai[173], Bahati[174], Nakuru West[175], Nakuru East[176], Westlands[274], Kilimani[275], Dagoretti[276], Langata[277], Kibra[278], Roysambu[279], Kasarani[280], Ruaraka[281], Embakasi South[282], Embakasi North[283], Embakasi Central[284], Embakasi East[285], Embakasi West[286], Makadara[287], Kamukunji[288], Starehe[289], Mathare[290]. |
| **Seasonal** | Tana River, Garissa, Wajir, Mandera, Marsabit, Isiolo, Meru, Tharaka-Nithi, Embu, Kitui, Turkana, Samburu, Elgeyo-Marakwet, Kajiado | Garsen[18], Galole[19], Bura[20], Dujis[27], Balambala[28], Lagdera[29], Dadaab[30], Fafi[31], Ijara[32], Wajir North[33], Wajir East[34], Tarbaj[35], Wajir West[36], Eldas[37], Wajir South[38], Mandera West[39], Banissa[40], Mandera North[41], Mandera South[42], Mandera East[43], Lafey[44], Moyale[45], North Horr[46], Saku[47], Laisamis[48], Isiolo North[49], Isiolo South[50], Igembe South[51], Igembe Central[52], Igembe North[53], Tigania West[54], Tigania East[55], North Imenti[56], Buuri[57], Central Imenti[58], South Imenti[59], Nithi[60], Maara[61], Tharaka[62], Manyatta[63], Runyenjes[64], Gachoka[65], Siakago[66], Mwingi North[67], Mwingi West[68], Mwingi East[69], Kitui West[70], Kitui Rural[71], Kitui Central[72], Kitui East[73], Kitui South[74], Turkana North[123], Turkana West[124], Turkana Central[125], Loima[126], Turkana South[127], Turkana East[128], Samburu Central[133], Samburu North[134], Samburu East[135], Marakwet East[147], Marakwet West[148], Keiyo North[149], Keiyo South[150], Kajiado North[183], Kajiado Central[184], Kajiado West[185], Kajiado East[186], Kajiado South[187]. |

**S2: Description of study covariates used in the model**

A non-spatial Poisson regression model was used to test the univariate and multivariate association between APOs its related risk factors, to identify the strength of each candidate covariate as a predictor and to identify the best subset of predictors for each outcome respectively. The Wald’s p-value, goodness of fit statistics and the associated confidence interval were assessed. Covariates significant at a p-value of <0.05 were then included in the space-time model.

**Table 2: Correlation analysis results**

| **Variable** | **Low-birth weight** | **Pre-term birth** | **Still births** | **Neonatal death** |
| --- | --- | --- | --- | --- |
| **Anc1** | 0.464 | 0.403 | 0.456 | 0.324 |
| **Anc4** | 0.446 | 0.375 | 0.406 | 0.224 |
| **Anaemia** | 0.302 | 0.254 | 0.304 | 0.112 |
| **LLITNs** | 0.380 | 0.333 | 0.313 | 0.217 |
| **IPT2** | 0.230 | 0.237 | 0.222 | 0.016 |
| **IPT1** | 0.212 | 0.236 | 0.181 | -0.009 |
| **Iron** | 0.191 | 0.137 | 0.148 | 0.090 |
| **Folate** | 0.142 | 0.123 | 0.153 | 0.106 |
| **Iron + Folate** | 0.087 | 0.056 | 0.104 | 0.023 |
| **Exercises** | 0.311 | 0.312 | 0.256 | 0.155 |
| **Breast-examined** | 0.054 | 0.052 | 0.067 | 0.033 |

**Table 3: Regression based on Wald’s P-values.**

| **­Variable** | Low-birth weight | Pre-term birth | Still births | Neonatal death |
| --- | --- | --- | --- | --- |
| Anc1 - | **0.000** | **0.000** | **0.000** | **0.000** |
| Anc4 (x1) | **0.000** | **0.013** | **0.000** | **0.024** |
| Anaemia (x2) | 0.466 | 0.126 | **0.002** | **0.019** |
| LLITNs (x3) | 0.030 | **0.007** | **0.019** | **0.036** |
| IPT2 (x4) | **0.000** | 0.423 | **0.000** | **0.000** |
| IPT1 | **0.000** | 0.761 | **0.000** | **0.000** |
| Iron (x5) | **0.000** | 0.863 | 0.276 | 0.599 |
| Folate (x6) | 0.074 | 0.411 | 0.092 | 0.035 |
| Iron + Folate | 0.538 | 0.281 | 0.942 | 0.272 |
| Exercises | 0.958 | 0.092 | 0.999 | 0.935 |
| Breast-examined | 0.747 | 0.613 | 0.105 | 0.251 |

|  | **2016** | **2017** | **2018** | **2019** |
| --- | --- | --- | --- | --- |
| **ANC4** |  |  |  |  |
| **Maternal anaemia** |  |  |  |  |
| **LLINs** |  |  |  |  |
| **IPT2** |  |  |  |  |
| **Iron** |  |  |  |  |
| **Folate** |  |  |  |  |

**Figure 1: Spatio-temporal distribution of covariates used in the joint model**

**Table 4: Risk of adverse pregnancy outcomes categorized by malaria endemic zones**

| **I. Low Birth Weight per 100 live births** | | |  |  |  |
| --- | --- | --- | --- | --- | --- |
| **Endemicity** | **2016** | **2017** | **2018** | **2019** | **Total** |
| Coast endemic | 5.6 (4.5 – 6.7) | 6.7 (5.8 – 7.7) | 7.8 (6.0 – 9.7) | 7.0 (6.0 – 8.0) | 6.8 (6.1 – 7.4) |
| Highland endemic | 5.1 (3.9 – 6.2) | 3.9 (3.3 – 4.6) | 4.5 (3.8 – 5.2) | 4.7 (3.9 – 5.5) | 4.6 (4.1 – 5.0) |
| Lake endemic | 3.4 (3.0 – 3.9) | 3.6 (3.1 – 4.1) | 3.9 (3.6 – 4.3) | 4.3 (3.8 – 4.9) | 3.8 (3.6 – 4.1) |
| Low risk | 4.6 (4.1 – 5.1) | 4.7 (4.2 – 5.1) | 5.2 (4.7 – 5.7) | 5.1 (4.6 – 5.6) | 4.9 (4.6 – 5.1) |
| Total | 4.3 (4.0 – 4.7) | 4.3 (4.0 – 4.6) | 4.8 (4.4 – 5.1) | 4.8 (4.5 – 5.1) | 4.5 (4.4 – 4.7) |
|  |  |  |  |  |  |
| **II. Pre-term Birth per 100 live births** | | | | | |
| Coast endemic | 2.7 (1.9 – 3.6) | 4.2 (1.9 – 6.5) | 2.9 (2.1 – 3.7) | 3.2 (2.1 – 4.2) | 3.2 (2.6 – 3.9) |
| Highland | 2.3 (1.7 – 2.9) | 1.9 (1.4 – 2.3) | 1.7 (1.4 – 2.1) | 2.1 (1.6 – 2.7) | 2.0 (1.8 – 2.3) |
| Lake endemic | 2.3 (1.8 – 2.8) | 2.8 (2.1 – 3.6) | 3.0 (2.3 – 3.7) | 3.1 (2.3 – 3.8) | 2.8 (2.5 – 3.1) |
| Low risk | 2.2 (1.7 – 2.7) | 2.5 (1.9 – 3.0) | 2.2 (1.8 – 2.5) | 2.3 (1.9 – 2.7) | 2.3 (2.1 – 2.5) |
| Seasonal | 1.7 (1.4 – 2.1) | 2.2 (1.4 – 2.9) | 1.8 (1.3 – 2.2) | 1.9 (1.5 – 2.3) | 1.9 (1.6 – 2.2) |
| Total | 2.2 (2.0 – 2.4) | 2.5 (2.2 – 2.8) | 2.2 (2.0 – 2.5) | 2.4 (2.1 – 2.6) | 2.3 (2.2 – 2.5) |
|  |  |  |  |  |  |
| **III. Stillbirths per 1000 live births** | | | | | |
| Coast endemic | 23.7 (18.2 – 29.2) | 26.9 (21.1 – 32.7) | 22.5 (17.8 – 27.1) | 22.8 (18.5 – 27.1) | 24.0 (21.5 – 26.4) |
| Highland | 23.2 (15.0 – 31.4) | 21.4 (16.1 – 26.7) | 18.1 (15.6 – 20.6) | 19.6 (15.9 – 23.4) | 20.6 (18.0 – 23.2) |
| Lake endemic | 16.0 (13.6 – 18.2) | 16.1 (13.8 – 18.5) | 16.0 (13.9 – 18.2) | 16.4 (14.3 – 18.6) | 16.1 (15.1 – 17.2) |
| Low risk | 15.0 (13.6 – 16.5) | 16.2 (14.5 – 18.0) | 16.0 (14.3 – 17.7) | 15.7 (14.3 – 17.1) | 15.7 (15.0 – 16.5) |
| Seasonal | 22.7 (19.3 – 26.2) | 21.4 (19.0 – 23.8) | 20.8 (18.5 – 23.1) | 19.6 (17.3 – 21.8) | 21.1 (19.8 – 22.4) |
| Total | 19.4 (17.4 – 21.3) | 19.3 (17.9 – 20.8) | 18.1 (17.0 – 19.1) | 18.1 (17.0 – 19.3) | 18.7(18.0 – 19.4) |
| **IV. Neonatal deaths per 1000 live births** | | | | | |
| Coast endemic | 7.1 (4.1 – 10.1) | 8.8 (4.2 – 13.4) | 7.9 (3.7 – 12.1) | 7.4 (3.5 -11.3) | 7.8 (5.9 – 9.7) |
| Highland | 7.3 (4.8 – 9.8) | 5.9 (3.9 – 7.8) | 6.1 (3.9 – 8.2) | 6.6 (4.2 – 8.9) | 6.4 (5.3 – 7.5) |
| Lake endemic | 6.7 (4.8 – 8.6) | 5.6 (4.3 – 7.0) | 6.7 (4.9 – 8.5) | 6.9 (5.1 – 8.7) | 6.5 (5.6 – 7.3) |
| Low risk | 7.8 (5.4 – 10.2) | 7.1 (4.9 – 9.3) | 7.8 (5.5 – 10.0) | 7.6 (5.1 – 10.1) | 7.6 (6.4 – 8.7) |
| Seasonal | 6.6 (5.0 – 8.1) | 6.7 (5.2 – 8.1) | 6.7 (5.1 -8.3) | 6.2 (4.8 – 7.6) | 6.5 (5.8 – 7.3) |
| Total | 7.1 (6.1 – 8.1) | 6.6 (5.7 -7.5) | 7.0 (6.0 – 8.0) | 6.9 (5.9 – 7.9) | 6.9 (6.4 – 7.4) |
